# Supplementary material for: Post-Fishing Ban Period: The Fish Diversity and Community Structure in the Poyang Lake Basin, Jiangxi Province, China
Source: Animals (Basel). 2025 Feb 4;15(3):433. doi: 10.3390/ani15030433 (PMC11815748; doi:10.3390/ani15030433)

Post-Fishing Ban Period: The Fish Diversity, Community Structure in the Poyang Lake Basin, Jiangxi Province, China

Chiping Kong [1], Yulan Luo [2,3], Qun Xu [1], Bao Zhang [1], Xiaoping Gao [1], Xianyong Wang [1], Zhen Luo [1], Zhengli Luo [2,3], Lekang Li [1,*] and Xiaoling Gong [2,3,*]

[1] Jiujiang Academy of Agricultural Sciences, Jiujiang 332000, China
[2] Key Laboratory of Exploration and Utilization of Aquatic Genetic Resources, Shanghai Ocean University, Ministry of Education, Shanghai 201306, China
[3] National Demonstration Center for Experimental Fisheries Science Education, Shanghai Ocean University, Shanghai 201306, China
* Correspondence: lekangli1987@126.com (L.L.); xlgong@shou.edu.cn (X.G.)

Table S1

List of fishes and ecological types in the Poyang Lake Basin (PLB).

Number	**Order**	Family	Genus	Species	Ecological type	Spring			Autumn		
						PYS	XBMS	XBUS	PYS	XBMS	XBUS
1	**Acipenseriformes**	Acipenseridae	*Acipenser*	*Acipenser schrenckii* ♀×*A.baerii* ♂	RS,C,LL	+			+		
2				*Acipenser sinensis*	RS,C,LL	+					
3	**Anguilliformes**	Anguillidae	*Anguilla*	*Anguilla japonica*	RS,C,UL	+					
4	**Beloniformes**	Hemiramphidae	*Hyporhamphus*	*Hyporhamphus intermedius*	L,O,UL				+	+	
5	**Clupeiformes**	Engraulidae	*Coilia*	*Coilia brachygnathus*	RL,C,UL	+			+		
6				*Coilia nasus*	RS,C,UL	+			+	+	
7	**Cypriniformes**	Balitoridae	*Vanmanenia*	*Vanmanenia pingchowensis*	R,O,DM			+			+
8		Catostominae	*Myxocyprinus*	*Myxocyprinus asiaticus*	RL,O,LL	+			+		
9		Cobitidae	*Botia*	*Botia almorhae*	R,O,DM				+		
10			*Cobitis*	*Cobitis macrostigma*	R,O,DM						+
11				*Cobitis rara*	R,O,DM	+					
12				*Cobitis sinensis*	R,O,DM	+		+	+		+
13			*Misgurnus*	*Misgurnus anguillicaudatus*	L,O,DM	+		+	+	+	+
14			*Parabotia*	*Parabotia banarescu*	R,O,DM		+				
15				*Parabotia fasciata*	R,C,DM	+	+		+		
16			*Paramisgurnus*	*Paramisgurnus dabryanus*	R,O,DM	+			+		
17			*Schistura*	*Schistura fasciolatus*	R,O,DM			+			
18				*Schistura incerta*	R,O,DM			+			+
19		Cyprinidae	*Abbottina*	*Abbottina rivularis*	R,O,DM	+	+	+	+		+
20			*Acheilognathus*	*Acheilognathus chankaensis*	L,O,LL		+		+	+	
21				*Acheilognathus gracilis*	L,O,LL			+			+
22				*Acheilognathus macropterus*	L,O,LL	+	+	+	+	+	+

23			*Acheilognathus tonkinensis*	L,O,LL						+
24			*Acheilognathus barbatulus*	R,C,LL			+			
25		*Acrossocheilus*	*Acrossocheilus fasciatus*	R,O,LL			+		+	+
26			*Acrossocheilus hemispinus*	R,O,LL						+
27			*Acrossocheilus kreyenbergii*	R,O,LL			+			+
28			*Acrossocheilus labiatus*	R,O,LL					+	+
29			*Acrossocheilus parallens*	R,O,LL			+			+
30		*Aristichthys*	*Aristichthys nobilis*	RL,P,UL	+	+	+	+	+	+
31		*Carassius*	*Carassius auratus*	L,O,DM	+	+	+	+	+	+
32		*Cirrhinus*	*Cirrhinus molitorella*	R,O,LL						+
33			*Cirrhinus mrigala*	R,O,LL				+		+
34		*Coreius*	*Coreius heterodon*	RL,O,DM	+			+		
35		*Ctenopharyngodon*	*Ctenopharyngodon idella*	RL,H,LL	+	+	+	+	+	+
36		*Culter*	*Culter alburnus*	R,C,UL	+	+	+	+	+	+
37			*Culter dabryi*	L,C,LL	+	+		+	+	
38			*Culter mongolicus*	R,C,UL	+	+	+	+	+	+
39			*Culter oxycephaloides*	R,O,UL	+	+		+	+	
40			*Culter oxycephalus*	R,C,LL	+	+		+	+	
41		*Cultrichthys*	*Cultrichthys erythropterus*	R,C,UL	+		+	+		+
42		*Cyprinus*	*Cyprinus carpio*	L,O,DM	+	+	+	+	+	+
43			*Cyprinus carpiovar*	R,O,LL	+	+				
44		*Distoechodon*	*Distoechodon tumirostris*	RL,O,LL			+		+	
45		*Elopichthys*	*Elopichthys bambusa*	RL,C,UL	+			+	+	
46		*Gnathopogon*	*Gnathopogon imberbis*	R,O,DM			+			+
47		*Hemibarbus*	*Hemibarbus labeo*	R,O,DM	+	+	+	+	+	+
48			*Hemibarbus maculatus*	L,C,LL	+	+		+	+	

49		*Hemiculter*	*Hemiculter bleekeri*	R,O,UL	+	+	+	+		+
50			*Hemiculter leucisculus*	RL,O,UL	+	+	+	+	+	
51		*Hypophthalmichthys*	*Hypophthalmichthys molitrix*	RL,P,UL	+	+	+	+	+	+
52		*Megalobrama*	*Megalobrama amblycephala*	L,H,LL	+		+	+		
53			*Megalobrama skolkovii*	L,O,LL	+	+	+	+	+	+
54		*Microphysogobio*	*Microphysogobio fukiensis*	R,O,LL			+			+
55		*Mylopharyngodon*	*Mylopharyngodon piceus*	RL,C,LL	+			+		
56		*Ochetobius*	*Ochetobius elongatus*	RL,C,LL	+					
57		*Opsariichthys*	*Opsariichthys bidens*	R,C,UL		+	+		+	+
58		*Parabramis*	*Parabramis pekinensis*	L,H,LL	+	+	+	+	+	
59		*Paracanthobrama*	*Paracanthobrama guichenoti*	L,O,LL	+			+		+
60		*Paracheilognathus*	*Paracheilognathus meridianus*	R,O,LL			+			+
61		*Pseudobrama*	*Pseudobrama simoni*	RL,O,LL	+	+	+	+	+	
62		*Pseudogobio*	*Pseudogobio vaillanti*	R,O,DM			+		+	+
63		*Pseudohemiculter*	*Pseudohemiculter dispar*	R,O,UL		+	+	+	+	+
64		*Pseudolaubuca*	*Pseudolaubuca sinensis*	L,O,UL	+	+	+	+	+	
65		*Pseudorasbora*	*Pseudorasbora parva*	L,O,UL	+		+	+	+	+
66		*Rhodeus*	*Rhodeus ocellatus*	L,O,DM	+	+	+	+	+	+
67			*Rhodeus sinensis*	R,O,LL	+			+		+
68		*Rhynchocypris*	*Rhynchocypris oxycephalus*	R,O,LL			+			+
69		*Sarcocheilichthys*	*Sarcocheilichthys kiangsiensis*	R,O,DM	+	+	+			
70			*Sarcocheilichthys nigripinnis*	R,O,UL	+			+		
71			*Sarcocheilichthys sinensis*	R,O,LL	+	+		+	+	
72		*Saurogobio*	*Saurogobio dabryi*	RL,O,LL	+	+	+	+	+	+
73			*Saurogobio dumerili*	R,O,UL	+			+	+	
74			*Saurogobio gymnocheilus*	R,O,DM	+					

75			*Sinibotia*	*Sinibotia superciliaris*	R,C,DM				+		
76			*Sinibrama*	*Sinibrama macrops*	R,O,LL	+	+	+		+	+
77			*Spinibarbus*	*Spinibarbus hollandi*	R,O,LL	+	+	+			+
78			*Squalidus*	*Squalidus argentatus*	RL,O,LL	+	+	+	+	+	+
79				*Squalidus atromaculatus*	R,O,DM						+
80				*Squalidus wolterstorffi*	R,O,LL			+			+
81			*Squaliobarbus*	*Squaliobarbus curriculus*	RL,O,LL		+	+	+	+	
82			*Xenocypris*	*Xenocypris argentea*	RL,O,LL	+	+		+	+	
83				*Xenocypris davidi*	R,O,DM	+	+	+	+	+	+
84				*Xenocypris microlepis*	R,O,LL			+			+
85			*Zacco*	*Zacco platypus*	R,O,LL			+			+
86	**Perciformes**	Channidae	*Channa*	*Channa argus*	L,C,DM	+	+	+	+	+	+
87				*Channa asiatica*	L,C,DM			+			+
88		Gobiidae	*Rhinogobius*	*Rhinogobius albomarginatus*	R,C,DM			+			+
89				*Rhinogobius duospilus*	R,C,DM			+			+
90				*Rhinogobius giurinus*	R,C,DM	+	+	+	+	+	+
91				*Rhinogobius rubspilus*	R,C,DM						+
92		Mastacembelidae	*Sinobdella*	*Sinobdella sinensis*	R,C,LL	+	+	+	+	+	
93		Odontobutidae	*Odontobutis*	*Odontobutis potamophila*	R,C,DM		+		+	+	
94		Serranidae	*Siniperca*	*Siniperca chuatsi*	L,C,DM	+	+		+	+	+
95				*Siniperca kneri*	R,C,DM	+	+		+	+	+
96				*Siniperca roulei*	R,C,LL	+	+	+	+		+
97				*Siniperca scherzeri*	R,C,DM	+	+	+	+	+	+
98				*Siniperca undulata*	R,C,DM		+	+			+
99	**Pleuronectiformes**	Soleidae	*Cynoglossus*	*Cynoglossus gracilis*	RS,O,DM	+			+		
100	**Salmoniformes**	Salangidae	*Protosalanx*	*Protosalanx hyalocranius*	RS,O,LL				+		

101	**Siluriformes**	Amblycipitidae	*Liobagrus*	*Liobagrus marginatus*	R,C,DM						+
102		Bagridae	*Hemibagrus*	*Hemibagrus macropterus*	R,C,DM	+		+	+		
103			*Leiocassis*	*Leiocassis argentivittatus*	R,O,LL	+			+		
104				*Leiocassis crassilabris*	R,C,DM	+			+		
105				*Leiocassis longirostris*	RL,C,DM	+	+	+	+	+	+
106			*Pelteobagrus*	*Pelteobagrus eupogon*	R,O,DM	+	+		+	+	
107				*Pelteobagrus fulvidraco*	L,O,DM	+	+	+	+	+	+
108				*Pelteobagrus nitidus*	L,O,DM				+	+	+
109				*Pelteobagrus vachelli*	R,O,DM	+	+	+	+	+	
110			*Pseudobagrus*	*Pseudobagrus albomarginatus*	R,O,DM	+	+	+	+		+
111				*Pseudobagrus ondan*	R,C,DM						+
112				*Pseudobagrus pratti*	L,O,DM	+					
113				*Pseudobagrus tenuis*	R,O,DM	+	+		+	+	+
114				*Pseudobagrus truncatus*	R,C,DM	+					
115			*Rhinogobio*	*Rhinogobio typus*	R,O,DM				+	+	+
116		Clariidae	*Clarias*	*Clarias fuscus*	R,C,DM	+					
117		Ictaluridae	*Ictalurus*	*Ictalurus punctatus*	R,C,DM		+			+	
118		Siluridae	*Silurus*	*Silurus asotus*	L,C,DM	+	+	+	+	+	+
119				*Silurus meridionalis*	R,C,DM	+		+	+		
120	**Synbranchiformes**	Synbranchidae	*Monopterus*	*Monopterus albus*	R,C,DM			+			

R: Riverine fish, L: Lake resident fish, RL: River-lake migratory fish, RS: River-sea migratory fish, H: Herbivorous, P: Planktivorous, C: Carnivorous, O: Omnivorous, UL: Upper-layer fish, LL: Lower-layer fish, DM: Demersal fish

Table S2

Diversity index of the PLB in spring and autumn.

Stations	Spring			Autumn		
	H'	D	J'	H'	D	J'
S1	2.829	3.551	0.743	2.827	3.543	0.756
S2	2.489	3.204	0.666	2.506	3.174	0.689
S3	2.847	3.611	0.752	2.254	3.512	0.607
S4	2.766	3.256	0.736	2.539	3.699	0.656
S5	2.818	3.833	0.720	2.277	3.245	0.613
S6	2.339	3.460	0.604	2.585	2.846	0.711
S7	2.121	2.612	0.601	1.751	2.592	0.496
S8	1.944	2.748	0.547	1.967	2.395	0.578
S9	1.883	1.925	0.601	1.762	2.228	0.535
S10	2.460	3.321	0.672	2.548	3.377	0.696
S11	2.577	3.075	0.699	2.196	2.791	0.608
S12	2.038	2.292	0.605	2.157	2.351	0.634
S13	2.063	2.582	0.590	2.186	2.696	0.620
S14	2.552	3.609	0.675	2.215	3.178	0.609
S15	2.292	1.502	0.923	0.771	1.035	0.371
S16	2.271	2.341	0.724	2.239	3.085	0.630
S17	2.803	4.041	0.771	2.212	2.860	0.696
S18	2.082	2.262	0.674	1.930	2.073	0.668
MAX	2.847	4.041	0.923	2.827	3.699	0.756
MIN	1.883	1.502	0.547	0.771	1.035	0.371
AVG.	2.399	2.957	0.683	2.162	2.816	0.621

Shannon-Wiener diversity index (H'), species richness index (D), and evenness index (J').

Table S3

Spring and autumn environmental factors in the PLB.

Season		Temp	pH	DO	TN	TP	Chl-a	MnO_4^-	NH_3	Oil	Cu
Spring	MAX	28.400	7.895	10.405	2.110	0.110	15.000	4.440	0.014	0.030	0.013
	MIN	19.800	6.780	7.050	0.510	0.005	1.000	0.660	0	0	0
	AVG.	23.261	7.279	8.677	1.488	0.052	4.639	1.858	0.003	0.014	0.002
Autumn	MAX	26.400	7.630	9.720	4.660	0.190	22.000	3.420	0.007	0.030	0.009
	MIN	20.500	6.710	7.050	0.120	0.010	3.000	0.569	0.001	0	0
	AVG.	22.861	7.164	8.531	1.399	0.056	9.167	1.703	0.002	0.016	0.004

Water temperature (Temp), pH, dissolved oxygen (DO), total nitrogen (TN), total phosphorus (TP), chlorophyll-a (Chl-a), permanganate index (MnO_4^-), ammonia (NH_3), oil (Oil), and copper (Cu).

Table S4

The body length-weight relationships of six endemic and typical species in the PLB.

Species	Areas	Body length (cm)			Body weight (g)			Body length-weight relationship	Number (ind)
		Average	Range	Dominant Group	Average	Range	Dominant Group		
Coilia brachygnathus	Yangtze River	22.20 ± 5.27	7.80 ~ 35.20	15 ~ 19 (24.68%)	43.30 ± 30.68	1.89 ~ 186.46	15 ~ 25 (20.81%)	$W=0.00213L^{3.14112}$ (R^2=0.8917)	1584
	Middle reaches	21.56 ± 5.03	12.00 ~ 36.20	17 ~ 23 (46.85%)	35.60 ± 27.41	4.80 ~ 163.04	15 ~ 25 (27.46%)	$W=0.00189L^{3.14523}$ (R^2=0.9561)	143
	Upper reaches	19.70 ± 3.75	9.00 ~ 36.10	17 ~ 21 (54.60%)	27.95 ± 18.62	2.01~ 142.61	15 ~ 25 (42.03%)	$W=0.00319L^{3.01755}$ (R^2=0.9135)	729
Coilia nasus	Yangtze River	30.65 ±3.30	22.51 ~ 41.00	29 ~ 31 (26.28%)	88.56 ± 32.68	10.12 ~ 232.78	70 ~ 90 (24.68%)	$W=0.00168L^{3.16472}$ (R^2=0.8560)	312
	Middle reaches	32.05 ± 3.78	16.80 ~ 38.50	31 ~ 33 (23.64%)	97.61 ± 34.42	14.53 ~ 162.61	50 ~ 90 (46.85%)	$W=0.00164L^{3.15906}$ (R^2=0.8699)	55
	Upper reaches	32.24 ± 4.16	3.20 ~ 39.90	31 ~ 35 (51.16%)	99.03 ± 33.76	7.10 ~ 227.89	90 ~ 130 (49.48%)	$W=0.00403L^{2.92357}$ (R^2=0.7677)	776
Pelteobagrus nitidus	Yangtze River	10.72 ± 1.65	3.20 ~ 25.94	9 ~ 13 (86.59%)	15.17 ± 11.32	0.77 ~ 271.10	12.5 ~17.5 (35.22%)	$W=0.00446L^{3.38158}$ (R^2=0.8987)	1445
	Middle reaches	11.97 ± 1.75	10.03 ~ 19.82	11 ~ 13 (80.33%)	20.99 ± 11.75	8.80 ~ 75.00	22.5 ~27.5 (29.51%)	$W=0.03354L^{2.57605}$ (R^2=0.8307)	61
	Upper reaches	10.11 ± 2.11	7.32 ~ 13.51	11 ~ 13 (30.77%)	14.26 ± 3.20	7.20 ~ 19.41	12.5 ~17.5 (61.54%)	$W=0.01011L^{3.20185}$ (R^2=0.9222)	13
	Poyang Lake	11.70 ± 2.26	1.72 ~ 28.38	11 ~ 13 (59.26%)	21.19 ± 22.04	1.59 ~248.22	12.5 ~17.5 (37.86%)	$W=0.012L^{2.99544}$ (R^2=0.9179)	486
Culter mongolicus	Yangtze River	23.18 ± 9.75	9.21 ~ 54.04	17.5 ~ 22.5 (32.95%)	258.81 ± 367.17	8.30 ~ 2369.88	70 ~90 (13.95%)	$W=0.00552L^{3.24592}$ (R^2=0.9777)	258
	Middle reaches	22.54 ± 6.83	10.18 ~ 57.02	22.5 ~ 27.5 (31.51%)	186.97 ± 224.79	11.81 ~ 2756.71	30 ~50 (13.50%)	$W=0.00549L^{3.2455}$ (R^2=0.9882)	310
	Upper reaches	24.80 ± 3.16	11.80 ~ 33.41	22.5 ~ 27.5 (79.73%)	184.41 ± 58.62	21.10 ~ 468.97	170 ~190 (28.38%)	$W=0.02253L^{2.79412}$ (R^2=0.8911)	74
	Poyang Lake	21.22 ± 8.82	10.10 ~ 50.94	12.5 ~ 17.5 (25.66%)	182.06 ± 300.22	10.38 ~ 2172.28	10 ~30 (26.11%)	$W=0.00329L^{3.36539}$ (R^2=0.9009)	226
Pelteobagrus vachelli	Yangtze River	14.92 ± 6.06	8.47 ~ 42.19	11 ~ 13 (33.42%)	69.09 ± 119.66	3.43 ~ 881.37	10 ~30 (56.44%)	$W=0.05245L^{2.55768}$ (R^2=0.8629)	365
	Middle reaches	22.64 ± 6.79	11.02 ~ 42.01	23 ~ 25 (23.08%)	191.18 ± 160.60	16.20 ~ 700.44	110 ~130 (11.54%)	$W=0.09089L^{2.41165}$ (R^2=0.9777)	27
	Upper reaches	17.97 ± 6.35	11.10 ~ 34.54	11 ~ 13 (26.32%)	106.62 ± 124.91	11.82 ~ 538.10	10 ~30 (31.58%)	$W=0.0144L^{2.97352}$ (R^2=0.9851)	19
	Poyang Lake	17.28 ± 7.04	6.00 ~ 42.41	11 ~ 13 (26.43%)	101.56 ± 126.47	1.50 ~ 706.50	10 ~30 (37.14%)	$W=0.05608L^{2.53734}$ (R^2=0.8565)	140
Megalobrama skolkovii	Yangtze River	21.35 ± 9.57	5.80 ~ 51.50	19.5 ~ 25.5 (38.51%)	343.12 ± 458.57	3.91 ~ 3246.41	0 ~ 25 (23.59%)<br>175 ~275 (27.92%)	$W=0.01368L^{3.13291}$ (R^2=0.9072)	623
	Middle reaches	21.46 ± 7.50	7.81 ~ 40.11	13.5 ~ 16.5 (29.21%)	284.96 ± 290.25	9.05 ~ 1443.40	50 ~ 70 (18.11%)	$W=0.02621L^{2.93245}$ (R^2=0.8844)	127

Upper reaches	17.56 ± 6.60	7.49 ~ 29.50	13.5 ~ 16.5 (45.00%)	165.82 ± 183.57	8.11 ~ 543.10	50 ~ 70 (35.00%)	W=0.01859L$^{3.04423}$ (R^2=0.9866)	20
Poyang Lake	26.12 ± 8.54	6.10 ~ 54.66	28.5 ~ 31.5 (22.62%)	471.06 ± 396.24	3.50 ~ 3895.00	0 ~25 (10.63%)<br>425 ~525 (15.61%)	W=0.01011L$^{3.20185}$ (R^2=0.9222)	884

Table S5

Differences in structural composition of fish communities in the PLB.

Sub-basin	*R*	*P*
PYS & XBMS	0.936	< 0.01
PYS & XBUS	0.986	< 0.01
XBMS & XBUS	0.688	< 0.05

Figure S1

Ecological niches of fishes in each sub-basin of PLB in different seasons.

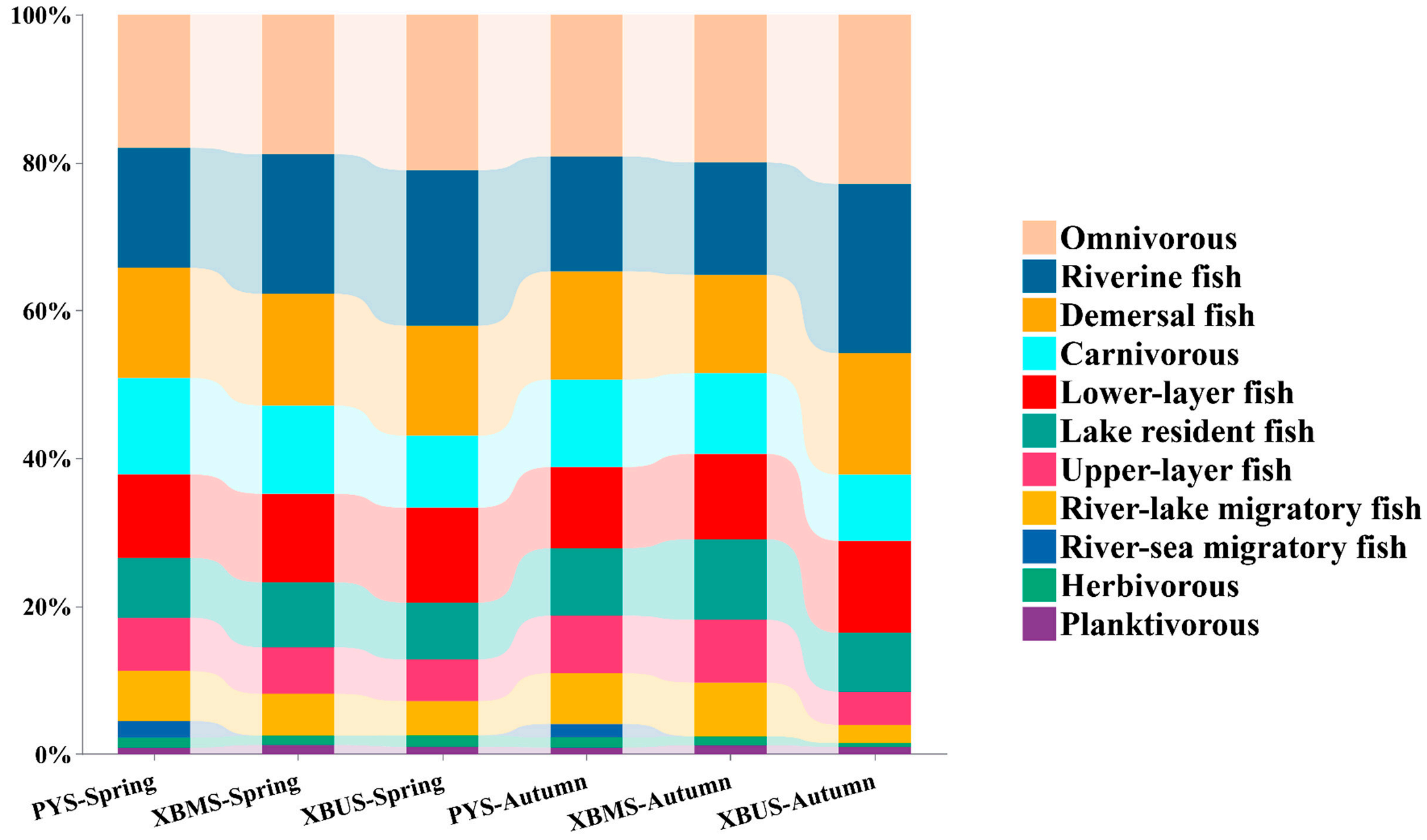

Supplement: Supplementary file 1 [file animals-15-00433-s001.zip › animals-3419632-supplementary.pdf]
